# Extracellular vimentin is an attachment factor that facilitates SARS-CoV-2 entry into human endothelial cells

**DOI:** 10.1073/pnas.2113874119

**Published:** 2022-01-25

**Authors:** Razie Amraei, Chaoshuang Xia, Judith Olejnik, Mitchell R. White, Marc A. Napoleon, Saran Lotfollahzadeh, Blake M. Hauser, Aaron G. Schmidt, Vipul Chitalia, Elke Mühlberger, Catherine E. Costello, Nader Rahimi

**Affiliations:** ^a^Department of Pathology, Boston University School of Medicine, Boston, MA 02118;; ^b^Center for Biomedical Mass Spectrometry, Boston University School of Medicine, Boston, MA 02118;; ^c^Department of Microbiology, Boston University School of Medicine, Boston, MA 02118;; ^d^National Emerging Infectious Diseases Laboratories, Boston University, Boston, MA 02118;; ^e^Renal Section, Department of Medicine, Boston University Medical Center, Boston, MA 02118;; ^f^Ragon Institute of Massachusetts General Hospital (MGH), Massachusetts Institute of Technology and Harvard University, Cambridge, MA 02139;; ^g^Department of Microbiology, Harvard Medical School, Boston, MA 02115;; ^h^Veterans Affairs Boston Healthcare System, Boston, MA 02118;; ^i^Institute of Medical Engineering and Sciences, Massachusetts Institute of Technology, Cambridge, MA 02139

**Keywords:** vimentin, SARS-CoV-2, viral entry, endothelial cells, ACE2

## Abstract

SARS-CoV-2 entry into host cells is a crucial step for virus tropism, transmission, and pathogenesis. Angiotensin-converting enzyme 2 (ACE2) has been identified as the primary entry receptor for SARS-CoV-2; however, the possible involvement of other cellular components in the viral entry has not yet been fully elucidated. Here we describe the identification of vimentin (VIM), an intermediate filament protein widely expressed in cells of mesenchymal origin, as an important attachment factor for SARS-CoV-2 on human endothelial cells. Using liquid chromatography–tandem mass spectrometry, we identified VIM as a protein that binds to the SARS-CoV-2 spike (S) protein. We showed that the S-protein receptor binding domain (RBD) is sufficient for S-protein interaction with VIM. Further analysis revealed that extracellular VIM binds to SARS-CoV-2 S-protein and facilitates SARS-CoV-2 infection, as determined by entry assays performed with pseudotyped viruses expressing S and with infectious SARS-CoV-2. Coexpression of VIM with ACE2 increased SARS-CoV-2 entry in HEK-293 cells, and shRNA-mediated knockdown of VIM significantly reduced SARS-CoV-2 infection of human endothelial cells. Moreover, incubation of A549 cells expressing ACE2 with purified VIM increased pseudotyped SARS-CoV-2-S entry. CR3022 antibody, which recognizes a distinct epitope on SARS-CoV-2-S-RBD without interfering with the binding of the spike with ACE2, inhibited the binding of VIM with CoV-2 S-RBD, and neutralized viral entry in human endothelial cells, suggesting a key role for VIM in SARS-CoV-2 infection of endothelial cells. This work provides insight into the pathogenesis of COVID-19 linked to the vascular system, with implications for the development of therapeutics and vaccines.

## Significance

Human angiotensin-converting enzyme 2 (ACE2) is the most widely known entry receptor for SARS-CoV-2. The possible involvement of other cellular components in viral entry mechanisms remains unknown. Vimentin is expressed in human endothelial cells, binds to SARS-CoV-2-spike, and expedites SARS-CoV-2 entry. Treatment of lung ACE2/A549 carcinoma cells with purified vimentin or coculture of ACE2/A549 cells with HEK-293 cells expressing vimentin increased ACE2-dependent viral entry. CR3022 antibody blocked vimentin interaction with SARS-CoV-2-spike and inhibited SARS-CoV-2 entry. Vimentin could facilitate SARS-CoV-2 infection and contribute to vascular complications associated with COVID-19.

Severe acute respiratory syndrome coronavirus 2 (SARS-CoV-2) is the causative agent of coronavirus disease 2019 (COVID-19) (1). SARS-CoV-2 binds to host receptors and attachment factors through its spike (S) glycoprotein and mediates membrane fusion and viral entry (2, 3). Although infection of cells along the respiratory tract was almost immediately defined as an important hallmark of the disease, the SARS-CoV-2 virus has also been detected not only in the lungs, but also in the cardiovascular system, brain, liver, kidneys, and intestine (4–7). Angiotensin-converting enzyme 2 (ACE2) is recognized as an important receptor for SARS-CoV-2 (1, 2). However, recent single-cell sequencing studies have demonstrated that ACE2 expression is relatively high in upper respiratory cells but low in the lower respiratory tract (8–12), and this result coupled with the identification of multiple other factors—including AXL receptor tyrosine kinase (11), Neuropilin-1 (13), CD209L/L-SIGN, CD209/DC-SIGN (14), and heparin sulfate (15)—as additional potential receptors or coreceptors for SARS-CoV-2, suggest that multiple receptors/coreceptors may facilitate SARS-CoV-2 entry in a cell type-dependent manner. Therefore, variations in the mechanisms of SARS-CoV-2 entry could account for its robust tropism, transmission, and pathogenesis.

To search for unidentified receptors involved in SARS-CoV-2 entry into endothelial cells, we used SARS-CoV-2 S-receptor binding domain (S-RBD) as bait and whole-cell lysate (WCL) of human umbilical vein endothelial cells (HUVEC-TERT) as a source for prey proteins followed by liquid chromatography–tandem mass spectrometry (LC-MS/MS) analysis of proteins that showed an affinity for the SARS-CoV-2 S-RBD. Our analysis identified vimentin (VIM) as a SARS-CoV-2 binding protein. Further biochemical and cell culture studies using pseudotyped viruses expressing SARS-CoV-2 S-protein or infectious SARS-CoV-2 demonstrated that VIM interacts with both SARS-CoV-2 S and ACE2 and acts as a coreceptor for SARS-CoV-2.

VIM is a type III intermediate filament protein and is widely expressed in cells of mesenchymal origin, such as endothelial cells, fibroblasts, and monocytes (16). In addition to its key role in intermediate filament formation, VIM is also present at the extracellular surface of endothelial cells and macrophages (17–19). Previous studies have found that extracellular VIM functions as an attachment factor or coreceptor for various viruses, including, SARS-CoV-1 (20), cowpea mosaic virus (21), Japanese encephalitis virus (22), dengue virus (23), and human papillomavirus (24). VIM could play an important role in promoting SARS-CoV-2 entry into the human vascular system, as well as other organs and tissues via *cis-* and *trans*-infection mechanisms.

## Results

### Identification of VIM as a SARS-CoV-2 S-Binding Protein.

**Fig. 1.**
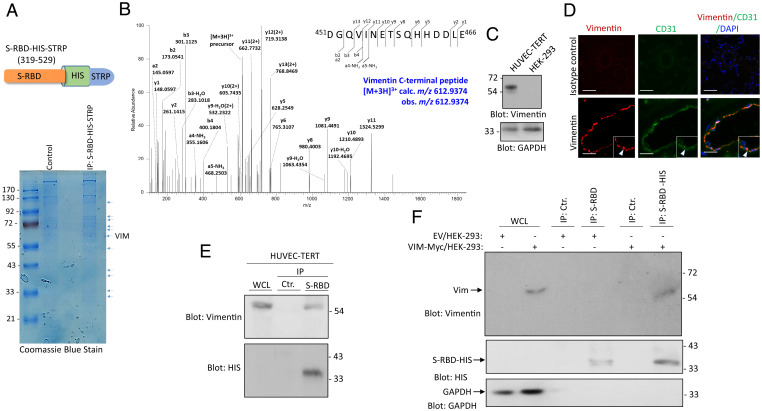
Identification of VIM as a SARS-CoV-2 S binding protein. (*A*) Schematic of SARS-CoV-2 S-RBD extended with a HIS and STRP. WCL from HUVEC-TERT cells were incubated with S-RBD-HIS-STRP-Ni-IMAG beads or Ni-IMAG beads alone and the captured proteins were resolved on SDS/PAGE and stained with Coomassie blue. The protein bands were cut out, subjected to in-gel digestion with trypsin, and the resulting peptide mixture was analyzed by LC-MS/MS. (*B*) HCD MS/MS spectrum is assigned to the C-terminal peptide of VIM, ^451^DGQVINETSQHHDDLE^466^ precursor [M + 3H]^3+^ calc. *m/z* 612.9374 obs. *m/z* 612.9374. Symbols: b_n_ = N-terminal fragments; y_n_ = C-terminal fragments. Peptide assignments were made by comparison to the Uniprot human database plus the sequence for the recombinant SARS-CoV-2 S-protein, using the Andromeda search engine with MaxQuant v1.6.14, and were checked manually. Seven VIM peptides, representing 14.4% of the protein amino acid sequence, were assigned with high confidence in the LC-MS/MS dataset. (*C*) Western blot analysis of VIM expression in HUVEC-TERT and control HEK-293 cells. The same membrane was blotted for GAPDH as a protein loading control. (*D*) PFA fixed human lung tissue was subjected to immunofluorescence staining. The tissue was stained with an anti-CD31 (endothelial marker) and anti-VIM antibodies. Image magnification: 50 µM. (*E*) WCL from HUVEC-TERT cells were incubated with S-RBD-HIS-STRP-Ni-IMAG beads or Ni-IMAG beads alone. The captured proteins were resolved on SDS/PAGE and blotted with an anti-VIM antibody. The same membrane was blotted with anti-HIS for S-RBD-HIS-STRP. (*F*) WCL from HEK-293 cells expressing control EV or VIM-Myc. The whole lysates were incubated with S-RBD-HIS-STRP-Ni-IMAG beads or Ni-IMAG beads alone and after extensive washing, and the captured proteins were resolved on SDS-PAGE and blotted with anti-VIM antibody. The same membrane was reblotted with anti-HIS for S-RBD-HIS-STRP or with GAPDH as the protein loading control.

SARS-CoV-2 infects both epithelial and mesenchymal cell types. However, the mechanisms by which SARS-CoV-2 infects mesenchymal cells, and the potential involvement of mediating factors, remain unknown. To identify mesenchymal cell-specific attachment factors or entry receptors for SARS-CoV-2, we employed SARS-CoV-2 S-RBD as bait and then used LC-MS/MS to analyze the proteins immunoprecipitated from HUVEC-TERT (Fig. 1*A*). VIM was identified as a candidate SARS-CoV-2 S-RBD binding protein (Fig. 1*B*). VIM is a 57-kDa intermediate filament protein expressed on multiple cell types with mesenchymal origin, including endothelial cells, fibroblasts, leukocytes, and others (25, 26). We confirmed the expression of VIM in HUVEC-TERT cells via Western blot analysis (Fig. 1*C*). Moreover, we showed that VIM is expressed in human pulmonary arterioles (Fig. 1*D*). Additional analysis of VIM expression in human lung tissue via publicly available single-cell RNA-sequence data (27) showed that VIM is highly expressed in human lung endothelial cells, macrophages, T cells, and granulocytes (*SI Appendix*, Fig. S1*A*). VIM is present at low levels in (type 1 and 2) and fibroblasts (*SI Appendix*, Fig. S1*A*). We also analyzed ACE2 expression for comparison. Consistent with the previous report (10), ACE2 is present only at relatively low levels in alveoli type 2 cells (*SI Appendix*, Fig. S1*B*). Moreover, we also examined expression of ACE2 in human lung tissue via immunofluorescence staining. We did not convincingly detect ACE2 in endothelial cells (only a faint staining was observed) (*SI Appendix*, Fig. S2).

To assess the binding of VIM to SARS-CoV-2 S-RBD, we used WCL from HUVEC-TERT cells and an anti-VIM antibody for immunoprecipitation, followed by Western blot analysis. The result demonstrated that SARS-CoV-2 S-RBD binds to VIM endogenously expressed in HUVEC-TERT cells (Fig. 1*E*). In an additional approach, we ectopically expressed VIM in HEK-293 cells and showed that SARS-CoV-2 S-RBD interacted with VIM overexpressed in HEK-293 cells (Fig. 1*F*). These data demonstrate that VIM binds to SARS-CoV-2 S-RBD, suggesting that it may facilitate SARS-CoV-2 entry.

### VIM Intermediate Filament Protein Is Present at the Extracellular Surface and Acts as an Attachment Factor for SARS-CoV-2.

**Fig. 2.**
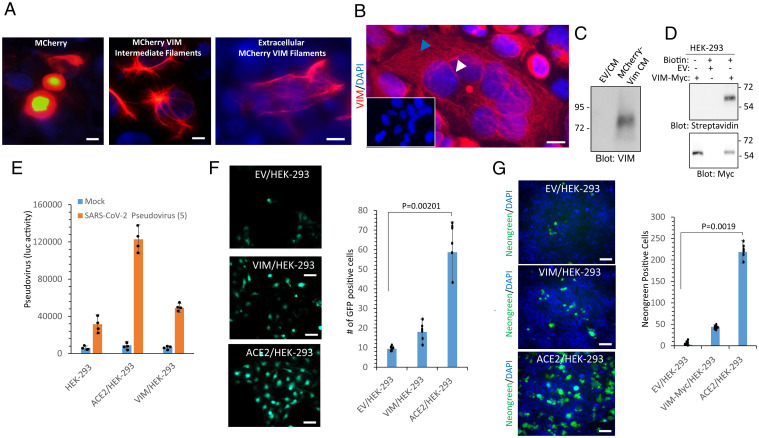
VIM intermediate filament is present on the extracellular surface and acts as an attachment factor for SARS-CoV-2. (*A*) HEK-293 cells were transfected with plasmids expressing mCherry or VIM-mCherry. Forty-eight hours after transfection, cells were fixed and the slides were viewed under a fluorescence microscope. Cell nuclei were stained with DAPI (blue). (Scale bars, 50 µm.) (*B*) HEK-293 cells expressing VIM were stained with an anti-VIM antibody and DAPI. The slides were viewed under a confocal microscope. (Scale bar, 50 µm; *Inset*, 50 µm.) (*C*) CM from HEK-293 cells transfected with an EV or a plasmid expressing VIM-mCherry were concentrated, resolved by SDS/PAGE, and subjected to Western blot analysis using an anti-VIM antibody. (*D*) HEK-293 cells expressing EV or VIM-Myc were subjected to cell surface biotinylation. Cells were lysed and cell lysates were immunoprecipitated with an anti-VIM antibody followed by Western blot analysis using Streptavidin antibody. The same membrane was stripped off the antibody and reblotted with anti-Myc antibody. (*E*) HEK-293 cells expressing EV, VIM or ACE2 were plated in 96-well plates (2.5 × 10^5^ cells per well, five wells per group). After overnight incubation, cells were treated with mock or SARS-CoV-2 S pseudovirus-luc and luciferase activity was measured after additional 24 h. (*F*) HEK-293 cells expressing EV, VIM, or ACE2 were plated in 96-well plates as described for *C*. Cells were treated with mock or SARS-CoV-2 S pseudovirus-GFP. After 48 h, cells were subjected to live cell imaging and pictures were taken from each well from random fields. GFP^+^ cells were counted. Graph is quantification of GFP^+^ cells (five well per group). (*G*) HEK-293 cells expressing EV, VIM-Myc, or ACE2 were seeded in 96-well plates (triplicate per group) and infected with SARS-CoV-2-mNG at an MOI of 2. After 24 h, cells were fixed in 10% neutral buffered formalin followed by staining with DAPI. Cells were viewed under a Nikon deconvolution fluorescence microscope and pictures were taken from random fields. Graph shows quantification of SARS-CoV-2-mNG^+^ cells (triplicates well per group, from two independent experiments). (Scale bars, 50 µm.)

VIM intermediate filaments are present within the cytoplasm and the on the extracellular surface of cells (19, 28). We confirmed the extracellular expression of VIM via ectopic expression of mCherry-VIM or VIM-Myc in HEK-293 cells. Live imaging of cells showed that mCherry-VIM is present both within and on the extracellular space of HEK-293 cells (Fig. 2*A*). A confocal microscopy analysis of VIM-Myc/HEK-293 cells also showed VIM containing intermediate filaments in the cytoplasm and membranous VIM (Fig. 2*B*). Furthermore, we collected conditioned media (CM) from HEK-293 cells expressing mCherry-VIM (cells were washed in media with low pH to remove the proteins that noncovalently are attached to the extracellular surface of HEK-293 cells), and after concentration, it was subjected to Western blot analysis. VIM was detected in the CM of cells expressing mCherry-VIM (Fig. 2*C*). In an alternative strategy, we also assessed the presence of extracellular VIM in HEK-293 cells expressing VIM-Myc via a cell surface biotinylation assay. VIM was found biotinylated in VIM/HEK-293 cells (Fig. 2*D*).

**Fig. 3.**
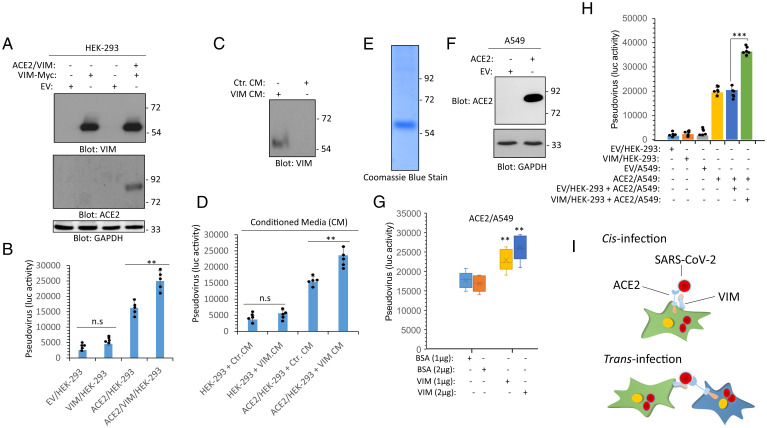
VIM binds to ACE2 and increases the binding of SARS-CoV-2 S-protein to ACE2. (*A*) WCL from HEK293 cells expressing an EV, VIM-Myc or coexpressing VIM-Myc with ACE2 were subjected to Western blot analysis using anti-VIM, anti-ACE2, or GAPDH antibodies. (*B*) HEK293 cells expressing EV, VIM-Myc, ACE2, or coexpressing VIM-Myc with ACE2 were plated in 96-well plates (2.5 × 10^5^ cells per well, five wells per group). After overnight incubation, cells were treated with mock or SARS-CoV-2 S pseudovirus-luc. After 24 h. cells were lysed and luciferase activity was measured. (*C*) Western blot analysis of CM from control HEK-293 cells or HEK-293 cells expressing VIM. (*D*) HEK-293 cells expressing EV or ACE2 were seeded in 96-well plates (2.5 × 10^5^ cells per well, five wells per group). Cells were treated with control CM (Ctr. CM) or CM from HEK-293 cells expressing VIM. After 1-h incubation, the media was removed and the cells were subjected to virus entry assay using SARS-CoV-2 S pseudovirus-luc. After 24 h, cells were lysed and luciferase activity was determined. cells expressing EV or VIM or ACE2 were plated in 96-well plates (2.5 × 10^5^ cells per well, five wells per group). After overnight incubation, cells were preincubated with purified VIM (1 or 2 µg per well) or the control vehicle (BSA). After 30 min, cells were washed with PBS to remove the unbound VIM. Cells then were treated with mock or SARS-CoV-2 S pseudovirus-luc and luciferase activity was measured after an additional 24 h. **P < 0.01, ***P < 0.001; n.s., not significant.

Next, we asked whether HEK-293 cells ectopically expressing VIM are permissive to infection with SARS-CoV-2 S pseudotyped virus as compared to HEK-293 cells expressing ACE2. The result showed that the expression of VIM in HEK-293 cells had a small effect on viral entry using luciferase (Fig. 2*E*) and GFP-based entry assays (Fig. 2*F*). Additionally, we subjected these cell lines to SARS-CoV-2 infection assay. Similarly, HEK-293 cells expressing VIM showed no significant increase in viral entry compared to HEK-293 cells expressing ACE2 (Fig. 2*G*). Then, we asked whether VIM could function as a coreceptor for SARS-CoV-2. To address this question, we used HEK-293 cells expressing ACE2 alone, VIM alone, or ACE2 and VIM together (Fig. 3*A*). We subjected these cells to S pseudotyped entry assays. The results demonstrated that coexpression of VIM with ACE2 significantly increased SARS-CoV-2 S-mediated entry in HEK-293 cells (*P* < 0.01) (Fig. 3*B*).

Next, we asked whether treatment of ACE2/HEK-293 cells with the extracellular VIM can increase SARS-CoV-2 entry. We collected CM containing cell surface proteins (i.e., cells were washed in media with low pH to remove the proteins noncovalently attached to the cell surface) from HEK-293 cells expressing empty vector (EV, control) or a plasmid expressing VIM. Western blot analysis confirmed the presence of VIM in the CM (Fig. 3*C*). The CM was concentrated and used in pseudotyped virus entry assays. Treatment of HEK-293 cells with CM containing VIM (VIM CM) had only a minor effect on viral entry. However, incubation of ACE2/HEK-293 cells with VIM CM markedly increased viral entry compared to ACE2/HEK cells in the absence of VIM CM (Fig. 3*D*). To make sure that VIM is responsible for increase in viral entry in the CM derived from HEK-293 expressing VIM, we purified VIM via affinity purification (Fig. 3*E*). Moreover, we generated a lung carcinoma cell line A549 expressing ACE2 (Fig. 3*F*). Treatment of ACE2/A549 cells with VIM increased the S pseudotyped entry in a dose-dependent manner (*P* < 0.01) (Fig. 3*G*). These data suggest that extracellular VIM acts as an attachment factor or coreceptor for SARS-CoV-2, as its coexpression with ACE2 or addition of exogenous VIM to ACE2-expressing cells, such as HEK-293 and A549 cells, increased viral entry.

### VIM Binds to a Distinct Epitope on SARS-CoV-2 S-RBD and Increases the Binding of SARS-CoV-2 S to ACE2.

**Fig. 4.**
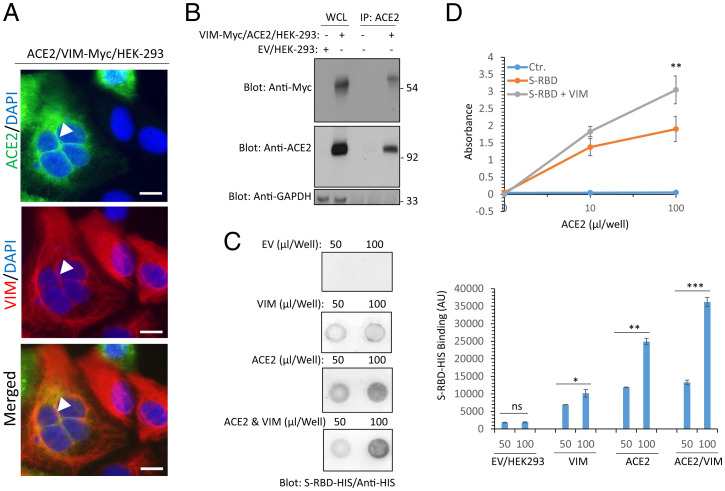
Extracellular VIM facilitates SARS-CoV-2 entry into human cells. (*A*) HEK-293 cells expressing ACE2 and VIM-Myc were costained with anti-ACE2, anti-VIM and DAPI. The slides were viewed by confocal microscopy. White arrowheads show colocalization of VIM with ACE2. (Scale bars, 50 µM.) (*B*) WCL from HEK-293 cells expressing control EV or coexpressing VIM-Myc with ACE2 were subjected to a coimmunoprecipitation assay using anti-ACE2 antibody. The immunoprecipitated proteins were analyzed by Western blot analysis using anti-Myc antibody for VIM. The same membrane was also probed for ACE2 and GAPDH. (*C*) Different concentrations of WCL from HEK-293 cells expressing EV, VIM, ACE2, or coexpressing VIM with ACE2 were blotted on the PVDF membrane. The membranes after blocking with BSA were incubated with S-RBD-HIS-STRP (1 µg/mL), followed with immunoblotting with anti-HIS antibody. Quantification of the blots is shown. (*D*) A 96-well plate coated with soluble ACE2 was incubated with RBD-HIS-STRP alone or RBD-HIS-STRP with CM containing VIM. The plate was subjected to ELISA and the binding of S-RBD with ACE2 determined with streptavidin-HRP. *P < 0.05, **P < 0.01, ***P < 0.001; ns, not significant.

We investigated the hypothesis that VIM interacts with ACE2 and increases the binding of SARS-CoV-2 S-protein with ACE2. To test this hypothesis, we asked whether VIM colocalizes with ACE2 in HEK-293 cells coexpressing VIM with ACE2. Our analysis showed that ACE2 is present in the membranous regions, particularly in cell–cell contact areas (Fig. 4*A*). VIM filaments are present at the cytoplasmic and extracellular compartments and colocalize with ACE2 at the cell–cell contact regions. Next, we performed a coimmunoprecipitation assay using lysates from HEK-293 cells coexpressing VIM and ACE2. The result showed that VIM was coimmunoprecipitated with ACE2 (Fig. 4*B*). Moreover, affinity-purified VIM also interacted with ACE2 expressed in HEK-293 cells (*SI Appendix*, Fig. S3). Next, we tested whether the binding of VIM to ACE2 could increase the ACE2 binding with SARS-CoV-2 S-protein. We performed dot blot assays where the cell lysates from HEK-293 cells express EV, VIM, ACE2 alone, or were coexpressed VIM with ACE2. After blocking with bovine serum albumin (BSA), the membrane was incubated with purified CoV-2 S-RBD streptavidin-binding peptide (STRP)-HIS protein and the binding of CoV-2 S-RBD-STRP-HIS with VIM and ACE2 was detected with anti-HIS antibody. The result showed that S-RBD-STRP-HIS protein bound more strongly to cell lysate derived from HEK-293 cells coexpressing VIM and ACE2 (Fig. 4*C*). Additionally, we used purified recombinant soluble ACE2 protein and measured the binding of S-RBD with ACE2 with or without VIM via ELISA. The result showed that the binding of S-RBD to ACE2 markedly increased when it was combined with VIM (Fig. 4*D*). This observation also suggests that the S-RBD site for binding with VIM is different from the S-RBD site for ACE2.

### VIM Binds to a Distinct Motif on SARS-CoV-2 S-RBD, Acts as an Attachment Factor for SARS-CoV-2, and Controls ACE2-Dependent Viral Entry.

**Fig. 5.**
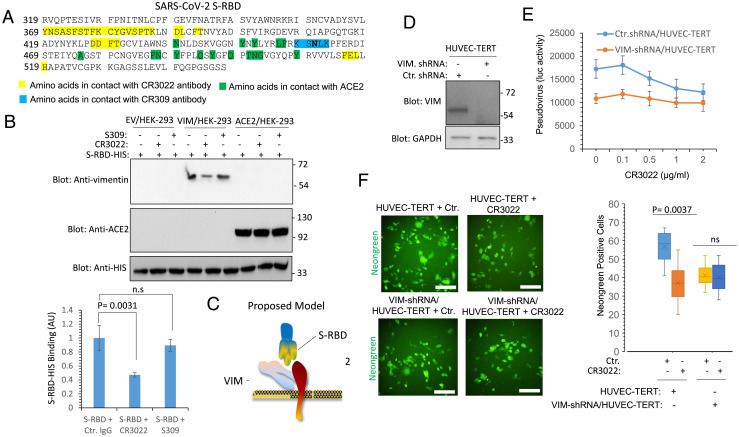
CR3022 antibody neutralizes SARS-CoV-2 entry in endothelial cells via VIM-dependent manner. (*A*) The amino acid sequence of SARS-CoV-2 S-RBD. Amino acids in highlighted in yellow are involved in interacting with CR3022 antibody. Amino acids highlighted in green are involved in interacting with ACE2. (*B*) S-RBD-HIS bound to Ni-IMAG beads (1 µg per group) incubated with an unrelated antibody, S309 antibody, or with CR3022 antibody. After 60-min incubation, the unbound antibodies were removed (washed 3× with PBS) and the S-RBD-HIS Ni-IMAG beads were incubated with HEK-293 cell lysates expressing EV, VIM, or ACE2. After extensive washing, the protein complexes were boiled and subjected to Western blot analysis using anti-VIM or anti-ACE2 antibodies. The graph is the average of three independent experiments. (*C*) Proposed model for binding of VIM with SARS-CoV-2 spike and ACE2. (*D*) Western blot analysis of HUVEC-TERT cells expressing control shRNA or VIM-shRNA. (*E*) HUVEC-TERT cells expressing control shRNA or VIM-shRNA were plated in 96-well plates (2.5 × 10^5^ cells per well, five wells per group). After overnight incubation cells were treated with mock orSARS-CoV-2 S pseudovirus-luc. However, before adding to cells, mock or SARS-CoV-2 S pseudovirus-luc particles were incubated with various concentrations of CR3022 antibody for 30 min at room temperature and then added to the cells. Luciferase activity was measured after additional 24 h. (*F*) HUVEC-TERT cells expressing control shRNA or VIM-shRNA were plated in 96-well plates (triplicate wells per group). The next day, SARS-CoV-2-mNG was preincubated CR3022 for 30 min before cells were infected with SARS-CoV-2-mNG at an MOI of 2. Twenty-four hours postinfection, the cells were fixed and analyzed by fluorescence microscopy. Three pictures per condition were taken from random fields. ns, not significant. (Scale bars, 50 µm.)

To map the potential interaction between SARS-CoV-2 S-RBD and VIM, we used a competition-based assay with antibodies with known molecular footprints on the RBD, namely CR3022 (29) and S309 (30). CR3022 binds the RBD outside receptor binding motif (RBM) within the RBD and does not directly compete with ACE2 (31) (Fig. 5*A*). S309 antibody also binds to RBD domain outside the RBM but it interacts with a conserved proteoglycan epitope and does not clash with ACE2 binding (30) (Fig. 5*A*). Preincubation of SARS-CoV-2 S-RBD with CR3022 but not with S309 decreased the binding of SARS-CoV-2 S-RBD with VIM, but not with ACE2 in Western blot analysis (Fig. 5*B*). This observation suggests that S-RBD uses two distinct motifs within RBD to engage VIM and ACE2 (Fig. 5*C*). Next, we questioned whether the CR3022 antibody can function as a neutralizing antibody in HUVEC-TERT. To demonstrate the requirement of VIM in the potential neutralizing activity of CR3022, we also knocked down VIM in HUVEC-TERT via short-hairpin RNA (shRNA) (Fig. 5*D*). Pseudotyped SARS-CoV-2-S and SARS-CoV-2 entry assays showed that preincubation of pseudotyped SARS-CoV-2-S or SARS-CoV-2 with CR3022 antibody inhibits cell entry in the parental HUVEC-TERT cells but not in VIM knocked down HUVEC-TERT cells (Fig. 5 *E* and *F*). Taken together, our data demonstrate the CR3022 antibody interferes with the binding of SARS-CoV-2-S and can function as a neutralizing antibody in relevant cell types when both VIM and ACE2 are expressed.

**Fig. 6.**
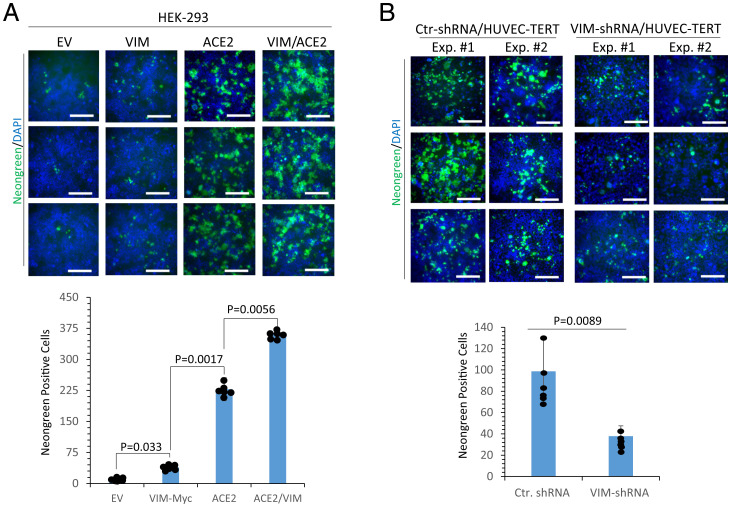
VIM is an attachment factor for SARS-CoV-2 and enhances ACE2-dependent viral entry. (*A*) HEK-293 cells expressing EV, VIM-Myc, ACE2, or coexpressing VIM-Myc with ACE2 were seeded in 96-well plates (triplicate per group). The next day, the cells were infected with SARS-CoV-2-mNG at an MOI of 2. Twenty-four hours postinfection, the cells were fixed, stained with DAPI, and analyzed by fluorescence microscopy. Three pictures per condition were taken from random fields. Graph is representative of SARS-CoV-2-mNG^+^ cells (triplicate well per group, two independent experiments). (*B*) HUVEC-TERT cells expressing control shRNA or VIM-shRNA were infected with SARS-CoV-2-mNG as described for *A*. Graph is representative of SARS-CoV-2-mNG^+^ cells (triplicate well per group, two independent experiments). (Scale bars, 50 µm.)

To gain further insight into the role of VIM in viral entry, we examined its role in ACE2-dependent SARS-CoV-2 infection. We infected HEK-293 cells expressing VIM alone or coexpressing VIM with ACE2 with SARS-CoV-2. HEK-293 cells coexpressing VIM with ACE2 were infected at a significantly higher rate than the cells expressing ACE2 alone (*P* = 0.0056, corresponding to two independent experiments, triplicates per group) (Fig. 6*A*). To address the role of endogenous VIM in facilitating SARS-CoV-2 infection of endothelial cells, we used VIM knocked out HUVEC-TERT cells and assessed SARS-CoV-2 infection rates. Knockdown of VIM significantly reduced infection of HUVEC-TERT cells compared to cells expressing control shRNA (*P* = 0.0089, two independent experiments, triplicates per group) (Fig. 6*B*). In conclusion, our data demonstrate that VIM is required for robust infection of endothelial cells by SARS-COV-2 and suggest that VIM could be a target for preventative or therapeutic intervention.

## Discussion

In this work, we provide evidence that VIM plays an important functional role in cell entry and infection by SARS-CoV-2. Extracellular VIM binds to SARS-CoV-2 S-RBD and acts as an attachment factor that facilitates viral entry and infection in endothelial cells. Although the lung is the primary target organ of SARS-CoV-2, there is growing indication that, in addition to the lung epithelial cells, SARS-CoV-2 also infects endothelial cells (14, 32, 33). Moreover, SARS-CoV-2 targets multiple other organs, including the cardiovascular system and other tissues, such as kidneys and intestine (34–37). Emerging evidence indicates that endothelial cells are direct targets of SARS-CoV-2 and that they also contribute to the pathogenesis of COVID-19 (7, 14, 32). Infection of endothelial cells, along with the inflammatory response induced by SARS-CoV-2, can lead to the activation of endothelial cells, inducing a proinflammatory and procoagulant state (38). Endothelial cell activation/dysfunction has been proposed to contribute to the pathology of COVID-19, ranging from vascular thrombosis (i.e., formation of a blood clot within a blood vessel), occlusion of alveolar capillaries, and altered angiogenesis to neurological symptoms (7, 14, 39). SARS-CoV-2–induced endothelial injury, along with other changes, converts normally anticoagulant endothelial cells to a procoagulant phenotype (40), leading to microvascular thrombosis and coagulopathy, and up-regulation of von Willebrand factor, all of which are clinical hallmarks of severe COVID-19 (41–43).

In the present study, we found that SARS-CoV-2 uses VIM as an attachment factor for entry into human endothelial cells. This conclusion is supported by a recently published report (posted in *bioRxiv*) on the involvement of VIM in SARS-CoV-2 infection showing that treatment with an anti- VIM antibody can block infection of HEK-293 cells expressing ACE2 (44). Our data suggest that SARS-CoV-2 S may bind both VIM and ACE2; VIM may serve as a bridge for S binding to ACE2, and this tertiary interaction enhances viral entry into human cells. Overexpression of VIM in HEK-293 cells only moderately enhanced SARS-CoV-2 infection, but either coexpression of VIM with ACE2 or treatment of HEK-293 cells expressing ACE2 with CM containing extracellular VIM significantly increased the level of infection of HEK-293 cells. Interestingly, the interaction site for the binding of S-RBD with VIM seems to differ from the location for its binding with ACE2. In two different assay systems (dot blot and ELISAs), VIM increased S-RBD binding with ACE2 (i.e., if the binding sites on S-RBD were the same for VIM and ACE2, increasing the amount of VIM would be expected to inhibit binding of S-RBD to ACE2).

Our data show that the CR3022 antibody recognizing an epitope on S-RBD not involved with ACE2 (31), also inhibited binding of SARS-CoV-2 S-RBD with VIM and inhibited viral entry in endothelial cells. The CR3022 antibody was originally identified from a convalescent plasma of SARS patient and is a neutralizing antibody that binds S-RBD of SARS-CoV and SARS-CoV-2 (31, 45) without interfering with spike binding with ACE2 (31). Our findings suggest that the neutralizing activity of the CR3022 antibody may be associated with blocking interaction with VIM, thereby inhibiting viral entry. However, previous studies demonstrated that treatment of kidney epithelial cells from the African green monkey cells (Vero cells), which is reported to express VIM (20, 46), with CR3022 antibody did not inhibit SARS-CoV-2 infection (31). Our previous study demonstrated that knockdown of ACE2 in endothelial cells only partially reduces SARS-CoV-2 infection (14), whereas knockdown of VIM in the same cells significantly reduced SARS-CoV-2 infection. This finding suggests that VIM may also interact with one or more other (as yet unidentified) receptors to mediate SARS-CoV-2 infection. Establishing the full range of the involvement of VIM in viral entry and infection will require further investigations.

VIM is widely expressed in human cells with mesenchymal origin, including endothelial cells, fibroblasts, macrophages, melanocytes, and lymphocytes. Additionally, VIM is expressed in alveolar type 2 cells and nasal goblet secretory cells, which are also positive for ACE2 (47, 48), and its expression is up-regulated in response to viral infection and inflammatory stimuli (49, 50), suggesting a potential role for VIM in infection of nasal and lung epithelial cells. In addition to its participation in infection, VIM also plays a critical role in lung inflammation and fibrosis through interaction with NLRP3 (NACHT, LRR, and PYD domains-containing protein 3) (51). Expression of ACE2 in endothelial cells (52) and lung alveolar cells is relatively low (*SI Appendix*, Figs. S1 and S2), but VIM as an attachment factor produced by mesenchymal cells (e.g., endothelial and fibroblasts) (53), could enhance the SARS-CoV-2 entry in respiratory cells via both *cis*- and *trans*-infection (44). Consistent with this idea, coculture of VIM/HEK-293 cells with ACE2/A549 cells increased viral entry (Fig. 3). Taken together, the observations we present may lead to the development of new antiviral therapeutics combining therapies that inhibit interactions of both ACE2 and VIM with SARS-CoV-2.

## Materials and Methods

### Antibodies, Plasmids, and shRNAs.

Anti-VIM antibody (cat# D21H3), anti-ACE2 antibody (Cat # 4355), and His-Tag antibody (cat# D3I1O) were purchased from Cell Signaling Technology. VIM shRNA (cat# sc-29522) was purchased from Santa Cruz Biotechnology. The shRNA plasmids used in this study are a pool of three to five lentiviral vector plasmids, each of which encodes a target specific 19 to 25 nt shRNA with a 6-bp loop. VIM mCherry plasmid (cat# 55158) was purchased from Addgene. VIM cDNA was further PCR-amplified and cloned into pQCXIP-Myc vector without the mCherry tag. SARS-CoV-2 S-RBD-HIS (GenBank: MN975262.1) was cloned into a pVRC vector containing a HRV 3C-cleavable C-terminal SBP-His8x tag). SARS-CoV-2 S-RBD was produced by transient transfection in mammalian Expi293F suspension cells, as previously described (14).

### S-Pseudotyped Lentivirus Production and Viral Entry Assay.

Nanoluc-expressing lentivirus vectors were generated via transient cotransfection of HEK-293T cells with pHAGE-Nanoluc, psPAX2 and SARS-COV-2 S/gp41 plasmids, as described previously (54). Virus containing cell supernatants were harvested 2 d posttransfection and filtered using a 0.45-µm syringe filters, aliquoted, and stored at −80 °C until further use (54). The p24^gag^ content of the virus stocks was quantified using a p24^gag^ ELISA, as described before (54). HEK293, VIM/HEK293 and ACE2/HEK-293 cells (2 × 10^4^ per well) were infected as described previously (54). Cells were lysed 48 h postinfection, and cell lysates were quantified for nanoluciferase activity.

### SARS-CoV-2 Neutralizing Antibody Development (CR3022 and S309).

Variable heavy- and light-chain amino acid sequences for the antibodies were obtained from the literature (30, 31). Sequences were codon optimized and synthesized (Integrated DNA Technologies). Variable heavy- and light-chain fragments were cloned into pVRC constructs, as previously described (55). The IgG heavy-chain vector contained an HRV 3C-cleavable 8xHis and SBP tags. HEK239F cells (ThermoFisher) were transfected per the manufacturer's protocol. Transfections were harvested after 5 d, centrifuged for clarification, and purified using immobilized metal affinity chromatography via the 8xHis tag with Cobalt-TALON resin (Takara) and eluded with imidazole followed with size-exclusion chromatography using a Superdex 200 Increase 10/300 GL column (GE Healthcare) in PBS.

### Propagation of SARS-CoV-2-mNG.

Recombinant SARS-CoV-2 expressing mNeonGreen (SARS-CoV-2-mNG) was kindly provided by Pey-Yong Shi, University of Texas Medical Branch, Galveston, TX, and the World Reference Center for Emerging Viruses and Arboviruses (56). This virus is based on SARS-CoV-2 isolate USA_WA1/2020. SARS-CoV-2-mNG stocks were grown in Vero E6 cells and virus titers were determined by tissue culture infectious dose 50 (TCID_50_) assay, as described previously (57). All work with SARS-CoV-2-mNG was performed in the biosafety level (BSL)-4 facility of the National Emerging Infectious Diseases Laboratories at Boston University following approved standard operating procedures.

### Infection of Cells with SARS-CoV-2 and Microscopic Analysis.

Cells (5 × 10^4^ per well) were seeded in 96-well plates. The next day, cells were infected with SARS-CoV-2-mNG at a multiplicity of infection (MOI) of 2. Generation of the SARS-CoV-2-mNG clone is as previously described (56). mNG is a tetrameric fluorescent protein derived from the cephalochordate *Branchiostoma lanceolatum* (58). One day postinfection, the cells were fixed in 10% neutral buffered formalin for at least 6 h at 4 °C and removed from the BSL-4 laboratory. Cell nuclei were stained with DAPI (Sigma-Aldrich) at 200 ng/mL diluted in blocking reagent (2% BSA, 0.2% Tween 20, 3% glycerin, and 0.05% NaN_3_ in PBS) for 1 h at room temperature in a light-protected box. Cells were washed with PBS and stored in PBS at 4 °C prior to imaging. Images of cells were acquired using a Nikon deconvolution microscope (20× magnification) equipped with camera.

### MS Analyses.

MS analyses of HUVEC-TERT immunoprecipitated proteins was carried out as described previously (59, 60). Briefly, nUPLC-MS/MS analyses were performed on an Orbitrap Fusion Lumos Tribrid mass spectrometer (ThermoFisher Scientific) coupled with an ACQUITY UPLC M-Class system (Waters) and a TriVersa NanoMate (Advion). For LC separation, a nanoEase Symmetry C18 UPLC Trap Column (100 Å, 5 μm, 180 μm × 20 mm; Waters) was used as the trapping column, and a nanoEase MZ HSS C18 T3 UPLC Column (100 Å, 1.8 μm, 75 μm × 100 mm; Waters) was used as the analytical column. The peptides were trapped at 4 μL/min for 4 min with 1% acetonitrile and 0.1% formic acid (Solvent A). Following the trapping step, peptides were separated on the analytical column according to the following conditions: 0 to 1 min: 2% B, 1 to 3 min: 2 to 5% B, 3 to 43 min: 5 to 40% B (Solvent B: 99% acetonitrile and 0.1% formic acid). All MS analyses were performed in the positive mode with the RF lens set to 30%. MS scans were acquired with the following settings: 120,000 resolution at *m/z* 200, scan range *m/z* 350 to 2,000, 1 μscan/MS, AGC target 1 × 10^6^, and a maximum injection time of 50 ms. For higher energy collision-induced dissociation (HCD) analyses, initial MS2 scans (NCE 30%) were acquired with the following settings: 15,000 resolution at *m/z* 200, scan range *m/z* 100 to 2,000, 1 μscan/MS, AGC target 1 × 10^6^, and a maximum injection time of 100 ms. MS/MS data were searched using the Andromeda search engine from MaxQuant v1.6.14. (Max Planck Institute of Biochemistry) The protein database (20,371 proteins) contained the entire UniProt human reference proteome (20,370 proteins) as downloaded on Oct. 29, 2020 and the protein sequence of FCS1 human recombinant COVID19 spike protein (1 protein).

### Cell Transfection and Retrovirus Production.

HEK-293 cells were grown in DMEM 10% FBS media to 60 to 70% confluency. VIM-mCherry plasmid or desired plasmids were transfected into HEK-293 cells via PEI (polyethylenimine). After 48 h, cells were lysed and subjected to coimmunoprecipitation or Western blotting as described in the figure legends. For retrovirus production, pQCXIP-Myc vector containing VIM or other cDNA of interest was transfected into 293-GPG cells, and viral supernatants were collected as previously described (61).

### Immunoprecipitation and Western Blot Analysis.

Cells were grown in 10-cm culture dishes until 80 to 90% confluence. Cells were lysed, and normalized WCL were subjected to immunoprecipitation by incubation with appropriate antibodies. Immunocomplexes were captured by incubation with protein A-Sepharose/protein G-agarose beads. After release via boiling the samples for 5 min at 95 °C, the immunoprecipitated proteins were subjected to Western blot analysis. In some instances, membranes were stripped by incubating them in a stripping buffer containing 6.25 mM Tris⋅HCl, pH 6.8, 2% SDS, and 100 mM β-mercaptoethanol at 50 °C for 30 min, washed in Western rinse buffer (20 mM Tris and 150 mM NaCl), and reprobed with the antibody of interest. The blots were scanned and subsequently quantified using ImageJ software (NIH).

### Dot Blot Assay.

Dot blots were carried out on a dot blot apparatus according to the manufacturer’s instructions. Briefly, a protein of interest was blotted on the PVDF membrane as indicated in the figure legends followed by standard Western blotting technique.

### Cell Surface Biotinylation Assay.

HEK 293 cells stably expressing VIM-Myc were subjected to cell surface biotinylation according to the manufacturer’s instructions (Pierce). Briefly, cells were biotinylated for 30 min at 4 °C and the reaction was stopped with quenching buffer. Cells were lysed and WCL was immunoprecipitated with the anti-VIM antibody. Biotinylated VIM was detected with blotting with streptavidin-HRP (SA-HRP).

### Affinity Purification of VIM.

Proteins from the cell lysates of HEK-293F cells expressing VIM-Myc were subjected to Spehadex-75 column chromatography, followed by an affinity purification using anti-VIM antibody.

### Statistical Analyses.

Experimental data were subjected to Student *t* test or one-way ANOVA, where appropriate, with representation from at least three independent experiments. *P* < 0.05 was considered significant.

## Supplementary Material

Supplementary File

## Data Availability

MS data are available via ProteomeXchange, http://www.proteomexchange.org/ (identifier PXD027595).

## References

[1] P. Zhou , A pneumonia outbreak associated with a new coronavirus of probable bat origin. Nature 579, 270–273 (2020). 32015507 10.1038/s41586-020-2012-7 PMC7095418

[2] M. Hoffmann , SARS-CoV-2 cell entry depends on ACE2 and TMPRSS2 and is blocked by a clinically proven protease inhibitor. Cell 181, 271–280.e8 (2020). 32142651 10.1016/j.cell.2020.02.052 PMC7102627

[3] J. Lan , Structure of the SARS-CoV-2 spike receptor-binding domain bound to the ACE2 receptor. Nature 581, 215–220 (2020). 32225176 10.1038/s41586-020-2180-5

[4] M. M. Lamers , SARS-CoV-2 productively infects human gut enterocytes. Science 369, 50–54 (2020). 32358202 10.1126/science.abc1669 PMC7199907

[5] V. G. Puelles , Multiorgan and renal tropism of SARS-CoV-2. N. Engl. J. Med. 383, 590–592 (2020). 32402155 10.1056/NEJMc2011400 PMC7240771

[6] L. Lin , Gastrointestinal symptoms of 95 cases with SARS-CoV-2 infection. Gut 69, 997–1001 (2020). 32241899 10.1136/gutjnl-2020-321013

[7] M. Ackermann , Pulmonary vascular endothelialitis, thrombosis, and angiogenesis in Covid-19. N. Engl. J. Med. 383, 120–128 (2020). 32437596 10.1056/NEJMoa2015432 PMC7412750

[8] S. Lukassen , SARS-CoV-2 receptor ACE2 and TMPRSS2 are primarily expressed in bronchial transient secretory cells. EMBO J. 39, e105114 (2020). 32246845 10.15252/embj.20105114 PMC7232010

[9] W. Sungnak , HCA Lung Biological Network, SARS-CoV-2 entry factors are highly expressed in nasal epithelial cells together with innate immune genes. Nat. Med. 26, 681–687 (2020). 32327758 10.1038/s41591-020-0868-6 PMC8637938

[10] X. Han , Construction of a human cell landscape at single-cell level. Nature 581, 303–309 (2020). 32214235 10.1038/s41586-020-2157-4

[11] S. Wang , AXL is a candidate receptor for SARS-CoV-2 that promotes infection of pulmonary and bronchial epithelial cells. Cell Res. 31, 126–140 (2021). 33420426 10.1038/s41422-020-00460-y PMC7791157

[12] Y. J. Hou , SARS-CoV-2 reverse genetics reveals a variable infection gradient in the respiratory tract. Cell 182, 429–446.e14 (2020). 32526206 10.1016/j.cell.2020.05.042 PMC7250779

[13] L. Cantuti-Castelvetri , Neuropilin-1 facilitates SARS-CoV-2 cell entry and infectivity. Science 370, 856–860 (2020). 33082293 10.1126/science.abd2985 PMC7857391

[14] R. Amraei , CD209L/L-SIGN and CD209/DC-SIGN act as receptors for SARS-CoV-2. ACS Cent. Sci. 7, 1156–1165 (2021). 34341769 10.1021/acscentsci.0c01537 PMC8265543

[15] T. M. Clausen , SARS-CoV-2 infection depends on cellular heparan sulfate and ACE2. Cell 183, 1043–1057.e15 (2020). 32970989 10.1016/j.cell.2020.09.033 PMC7489987

[16] J. Lowery, E. R. Kuczmarski, H. Herrmann, R. D. Goldman, Intermediate filaments play a pivotal role in regulating cell architecture and function. J. Biol. Chem. 290, 17145–17153 (2015). 25957409 10.1074/jbc.R115.640359 PMC4498054

[17] T. Päll , Soluble CD44 interacts with intermediate filament protein vimentin on endothelial cell surface. PLoS One 6, e29305 (2011). 22216242 10.1371/journal.pone.0029305 PMC3244446

[18] Y. Zou, L. He, S.-H. Huang, Identification of a surface protein on human brain microvascular endothelial cells as vimentin interacting with *Escherichia coli* invasion protein IbeA. Biochem. Biophys. Res. Commun. 351, 625–630 (2006). 17083913 10.1016/j.bbrc.2006.10.091

[19] N. Mor-Vaknin, A. Punturieri, K. Sitwala, D. M. Markovitz, Vimentin is secreted by activated macrophages. Nat. Cell Biol. 5, 59–63 (2003). 12483219 10.1038/ncb898

[20] Y. T.-C. Yu , Surface vimentin is critical for the cell entry of SARS-CoV. J. Biomed. Sci. 23, 14 (2016). 26801988 10.1186/s12929-016-0234-7 PMC4724099

[21] K. J. Koudelka , Endothelial targeting of cowpea mosaic virus (CPMV) via surface vimentin. PLoS Pathog. 5, e1000417 (2009). 19412526 10.1371/journal.ppat.1000417 PMC2670497

[22] S. Das, V. Ravi, A. Desai, Japanese encephalitis virus interacts with vimentin to facilitate its entry into porcine kidney cell line. Virus Res. 160, 404–408 (2011). 21798293 10.1016/j.virusres.2011.06.001

[23] J. Yang , Superficial vimentin mediates DENV-2 infection of vascular endothelial cells. Sci. Rep. 6, 38372 (2016). 27910934 10.1038/srep38372 PMC5133558

[24] G. Schäfer , Vimentin modulates infectious internalization of human papillomavirus 16 pseudovirions. J. Virol. 91, e00307-17 (2017). 28566373 10.1128/JVI.00307-17 PMC5533935

[25] J. M. Dave, K. J. Bayless, Vimentin as an integral regulator of cell adhesion and endothelial sprouting. Microcirculation 21, 333–344 (2014). 24387004 10.1111/micc.12111

[26] R. M. Evans, Vimentin: The conundrum of the intermediate filament gene family. BioEssays 20, 79–86 (1998). 9504050 10.1002/(SICI)1521-1878(199801)20:1<79::AID-BIES11>3.0.CO;2-5

[27] M. Uhlén , Proteomics. Tissue-based map of the human proteome. Science 347, 1260419 (2015). 25613900 10.1126/science.1260419

[28] B. Xu , The endothelial cell-specific antibody PAL-E identifies a secreted form of vimentin in the blood vasculature. Mol. Cell. Biol. 24, 9198–9206 (2004). 15456890 10.1128/MCB.24.20.9198-9206.2004 PMC517872

[29] X. Tian , Potent binding of 2019 novel coronavirus spike protein by a SARS coronavirus-specific human monoclonal antibody. Emerg. Microbes Infect. 9, 382–385 (2020). 32065055 10.1080/22221751.2020.1729069 PMC7048180

[30] D. Pinto , Cross-neutralization of SARS-CoV-2 by a human monoclonal SARS-CoV antibody. Nature 583, 290–295 (2020). 32422645 10.1038/s41586-020-2349-y

[31] M. Yuan , A highly conserved cryptic epitope in the receptor binding domains of SARS-CoV-2 and SARS-CoV. Science 368, 630–633 (2020). 32245784 10.1126/science.abb7269 PMC7164391

[32] Z. Varga , Endothelial cell infection and endotheliitis in COVID-19. Lancet 395, 1417–1418 (2020). 32325026 10.1016/S0140-6736(20)30937-5 PMC7172722

[33] V. Monteil , Inhibition of SARS-CoV-2 infections in engineered human tissues using clinical-grade soluble human ACE2. Cell 181, 905–913.e7 (2020). 32333836 10.1016/j.cell.2020.04.004 PMC7181998

[34] W. J. Wiersinga, A. Rhodes, A. C. Cheng, S. J. Peacock, H. C. Prescott, Pathophysiology, transmission, diagnosis, and treatment of coronavirus disease 2019 (COVID-19): A review. JAMA 324, 782–793 (2020). 32648899 10.1001/jama.2020.12839

[35] J. A. Fried , The variety of cardiovascular presentations of COVID-19. Circulation 141, 1930–1936 (2020). 32243205 10.1161/CIRCULATIONAHA.120.047164 PMC7314498

[36] M. Arentz , Characteristics and outcomes of 21 critically Ill patients with COVID-19 in Washington State. JAMA 323, 1612–1614 (2020). 32191259 10.1001/jama.2020.4326 PMC7082763

[37] S. Richardson , the Northwell COVID-19 Research Consortium, Presenting characteristics, comorbidities, and outcomes among 5700 patients hospitalized with COVID-19 in the New York City area. JAMA 323, 2052–2059 (2020). 32320003 10.1001/jama.2020.6775 PMC7177629

[38] R. Amraei, N. Rahimi, COVID-19, renin-angiotensin system and endothelial dysfunction. Cells 9, 1652 (2020). 10.3390/cells9071652 PMC7407648 32660065

[39] M. H. Lee , Microvascular injury in the brains of patients with Covid-19. N. Engl. J. Med. 384, 481–483 (2021). 33378608 10.1056/NEJMc2033369 PMC7787217

[40] N. Rahimi, Defenders and challengers of endothelial barrier function. Front. Immunol. 8, 1847 (2017). 29326721 10.3389/fimmu.2017.01847 PMC5741615

[41] J. K. Liao, Linking endothelial dysfunction with endothelial cell activation. J. Clin. Invest. 123, 540–541 (2013). 23485580 10.1172/JCI66843 PMC3561809

[42] D. R. J. Arachchillage, M. Laffan, Abnormal coagulation parameters are associated with poor prognosis in patients with novel coronavirus pneumonia. J. Thromb. Haemost. 18, 1233–1234 (2020). 32291954 10.1111/jth.14820 PMC7262191

[43] N. Tang, D. Li, X. Wang, Z. Sun, Abnormal coagulation parameters are associated with poor prognosis in patients with novel coronavirus pneumonia. J. Thromb. Haemost. 18, 844–847 (2020). 32073213 10.1111/jth.14768 PMC7166509

[44] L. Suprewicz , Extracellular vimentin as a target against SARS-CoV-2 host cell invasion. *Small*, 10.1002/smll.202105640 (2021). PMC9252327 34866333

[45] J. ter Meulen , Human monoclonal antibody combination against SARS coronavirus: Synergy and coverage of escape mutants. PLoS Med. 3, e237 (2006). 16796401 10.1371/journal.pmed.0030237 PMC1483912

[46] N. Du , Cell surface vimentin is an attachment receptor for enterovirus 71. J. Virol. 88, 5816–5833 (2014). 24623428 10.1128/JVI.03826-13 PMC4019121

[47] C. G. K. Ziegler , HCA Lung Biological Network. Electronic address: lung-network@humancellatlas.org; HCA Lung Biological Network, SARS-CoV-2 receptor ACE2 is an interferon-stimulated gene in human airway epithelial cells and is detected in specific cell subsets across tissues. Cell 181, 1016–1035.e19 (2020). 32413319 10.1016/j.cell.2020.04.035 PMC7252096

[48] M. Nishioka , Fibroblast-epithelial cell interactions drive epithelial-mesenchymal transition differently in cells from normal and COPD patients. Respir. Res. 16, 72 (2015). 26081431 10.1186/s12931-015-0232-4 PMC4473826

[49] A. Lilienbaum, D. Paulin, Activation of the human vimentin gene by the Tax human T-cell leukemia virus. I. Mechanisms of regulation by the NF-kappa B transcription factor. J. Biol. Chem. 268, 2180–2188 (1993). 8420985

[50] S. Ozden , Muscle wasting induced by HTLV-1 tax-1 protein: An in vitro and in vivo study. Am. J. Pathol. 167, 1609–1619 (2005). 16314474 10.1016/S0002-9440(10)61245-X PMC1613204

[51] G. dos Santos , Vimentin regulates activation of the NLRP3 inflammasome. Nat. Commun. 6, 6574 (2015). 25762200 10.1038/ncomms7574 PMC4358756

[52] N. Rahimi, C-type lectin CD209L/L-SIGN and CD209/DC-SIGN: Cell adhesion molecules turned to pathogen recognition receptors. Biology (Basel) 10, 1 (2020). 33375175 10.3390/biology10010001 PMC7822156

[53] G. Mansueto, COVID-19: Brief check through the pathologist’s eye (autopsy archive). Pathol. Res. Pract. 216, 153195 (2020). 32890939 10.1016/j.prp.2020.153195 PMC7452828

[54] H. Akiyama , HIV-1 intron-containing RNA expression induces innate immune activation and T cell dysfunction. Nat. Commun. 9, 3450 (2018). 30150664 10.1038/s41467-018-05899-7 PMC6110775

[55] A. G. Schmidt , Viral receptor-binding site antibodies with diverse germline origins. Cell 161, 1026–1034 (2015). 25959776 10.1016/j.cell.2015.04.028 PMC4441819

[56] X. Xie , An infectious cDNA clone of SARS-CoV-2. Cell Host Microbe 27, 841–848.e3 (2020). 32289263 10.1016/j.chom.2020.04.004 PMC7153529

[57] J. Huang , SARS-CoV-2 infection of pluripotent stem cell-derived human lung alveolar type 2 cells elicits a rapid epithelial-intrinsic inflammatory response. Cell Stem Cell 27, 962–973.e7 (2020). 32979316 10.1016/j.stem.2020.09.013 PMC7500949

[58] N. C. Shaner , A bright monomeric green fluorescent protein derived from *Branchiostoma lanceolatum*. Nat. Methods 10, 407–409 (2013). 23524392 10.1038/nmeth.2413 PMC3811051

[59] Y. H. W. Wang , IGPR-1 is required for endothelial cell-cell adhesion and barrier function. J. Mol. Biol. 428, 5019–5033 (2016). 27838321 10.1016/j.jmb.2016.11.003 PMC5138093

[60] E. J. Hartsough , Lysine methylation promotes VEGFR-2 activation and angiogenesis. Sci. Signal. 6, ra104 (2013). 24300896 10.1126/scisignal.2004289 PMC4108444

[61] N. Rahimi, V. Dayanir, K. Lashkari, Receptor chimeras indicate that the vascular endothelial growth factor receptor-1 (VEGFR-1) modulates mitogenic activity of VEGFR-2 in endothelial cells. J. Biol. Chem. 275, 16986–16992 (2000). 10747927 10.1074/jbc.M000528200

