# Supplementary material for: Extracellular vimentin is an attachment factor that facilitates SARS-CoV-2 entry into human endothelial cells
Source: Proc Natl Acad Sci U S A. 2022 Jan 25;119(6):e2113874119. doi: 10.1073/pnas.2113874119 (PMC8833221; doi:10.1073/pnas.2113874119)

Supplemental Data

Extracellular Vimentin is an Attachment Factor that Facilitates SARS-CoV-2 Entry into Human Endothelial Cells

Razie Amraei[1], Chaoshuang Xia[2], Judith Olejnik[3,4], Mitchell R White[3,4], Marc A. Napoleon[5], Blake M. Hauser[6,7], Aaron Schmidt[6,7], Vipul Chitalia[5], Elke Mühlberger*[3,4], Catherine E Costello*[2], Nader Rahimi*[1]

[1]Department of Pathology, School of Medicine, Boston University Medical Campus, Boston, MA 02118.
[2]Center for Biomedical Mass Spectrometry, Boston University School of Medicine, Boston, MA 02118
[3]Department of Microbiology, Boston University School of Medicine, Boston, MA 02118.
[4]National Emerging Infectious Diseases Laboratories (NEIDL), Boston University, Boston, MA.
[5]Renal Section, Department of Medicine, Boston University Medical Center, Boston, MA.
[6]Ragon Institute of MGH, MIT, and Harvard, Cambridge, MA 02139. [7]Department of Microbiology, Harvard Medical School, Boston, MA 02115.

Running Title: Vimentin is an Attachment Factor for SARS-CoV-2
Conflict of Interest: Authors declare no conflict of interest.
Key Words: SARS-CoV-2, Vimentin, attachment factor, SARS-CoV-2 receptor, spike protein, RBD domain, endothelial cells, mesenchymal cells.

Corresponding authors:
Catherine E Costello
cecmsms@bu.edu
Nader Rahimi
nrahimi@bu.edu

Acknowledgment: This work was supported in part through a grant from BUSM Genome Science Institute (NR and CEC); NIH grants R24 GM134210 and S10 OD021728 (to CEC), funding from BUSM COVID-19 ARC (to CEC and NR) and BUSM Clinical & Translational Science Institute awards from NIH NCATS grant UL1TR001430 (to EM and CEC). Fast Grants (EM), the Evergrande COVID-19 Response Fund Award from the Massachusetts Consortium on Pathogen Readiness (EM and AGS). NIH R01 AI146779 (AGS) and NIGMS T32 GM007753 (AGS). The content is solely the responsibility of the authors and does not necessarily represent the official views of the funding agencies. The authors thank Ellen L. Suder, Boston University, for technical assistance.

S. Figure 1: **Vimentin and ACE2 expression in human lung tissue**. (**A &B**)) Single cell RNA sequence data showing expression of vimentin and ACE2. The data extracted from the Human Protein Atlas (https://www.proteinatlas.org/) on 02-25-2021.

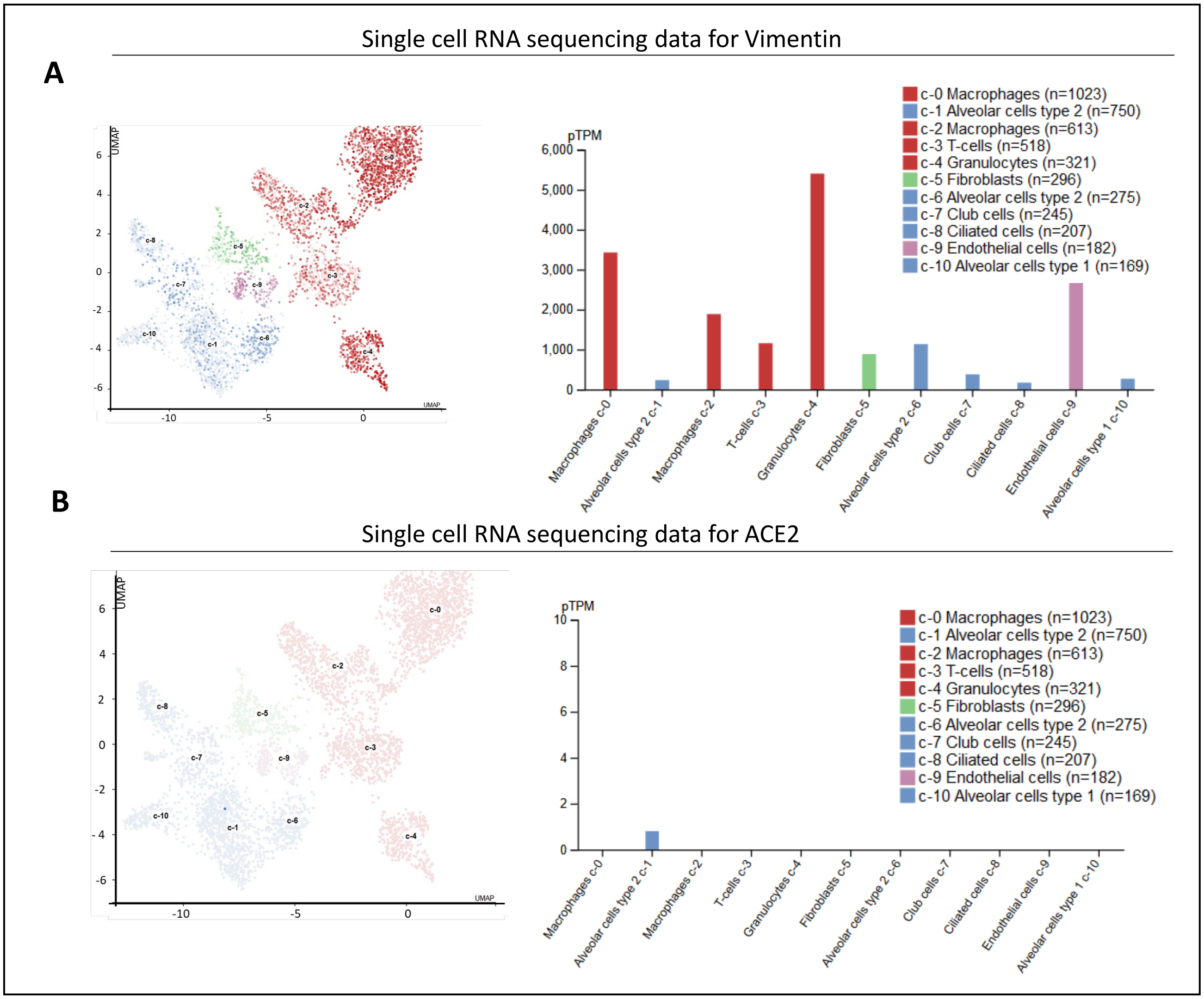

S. Figure 2. Expression of ACE2 in human lung tissue. PFA fixed human lung tissues were subjected to immunofluorescence staining. Lung tissue stained with a control IgG (**A**) or with an anti-ACE2 antibody (**B**). Image magnification 40X.

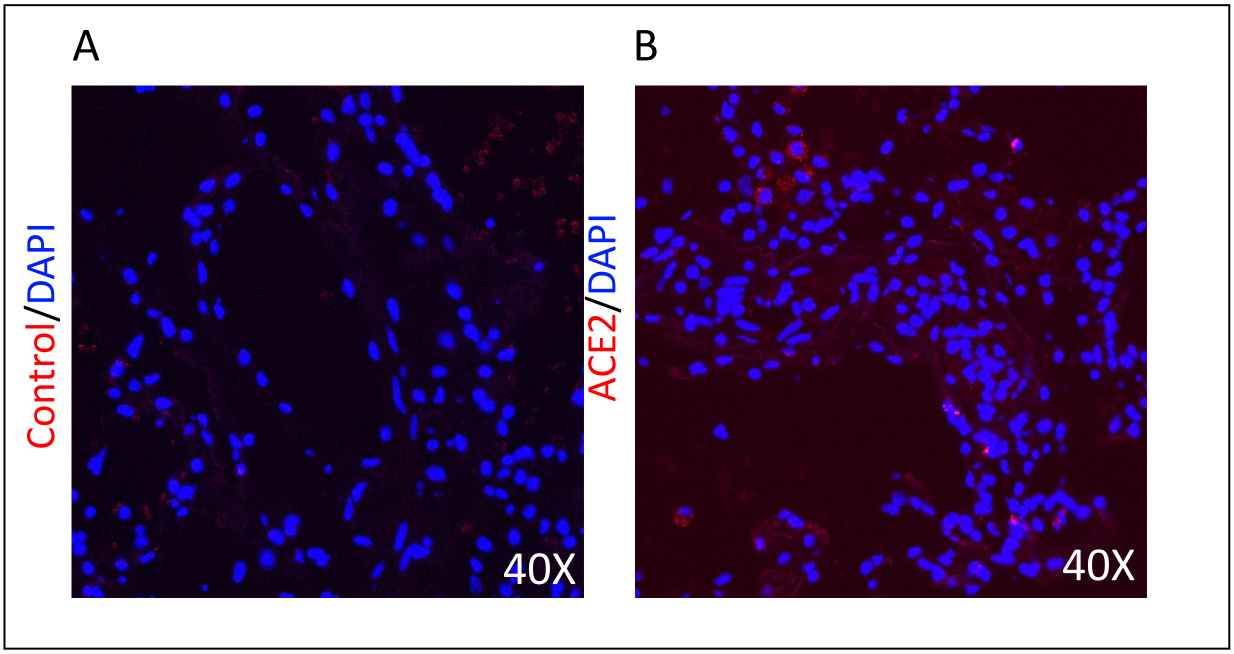

S. Figure 3. Affinity purified vimentin binds to ACE2. Affinity purified vimentin-myc (1µg/group) was incubated with the cell lysate of HEK-293 cells expressing ACE2, followed by immunoprecipitation with an anti-Myc antibody and immunoblotting with an anti-ACE2 antibody. The same membrane was blotted with an anti-Myc antibody.

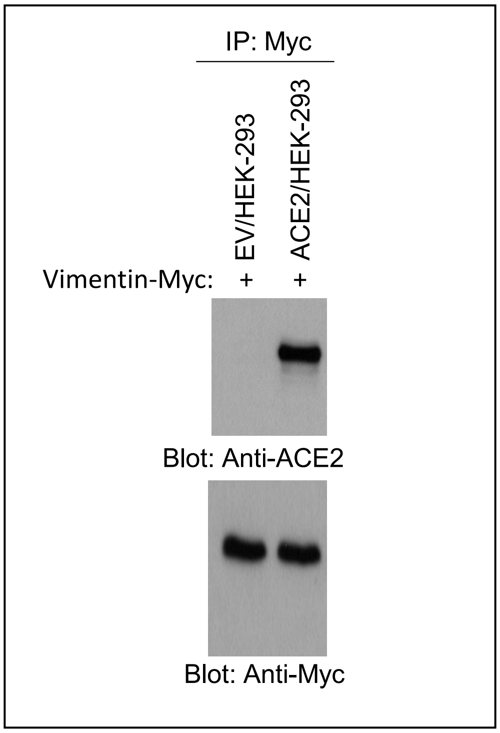

Supplement: Supplementary File [file pnas.2113874119.sapp.pdf]
